# Dissociation of Progressive Dopaminergic Neuronal Death and Behavioral Impairments by Bax Deletion in a Mouse Model of Parkinson's Diseases

**DOI:** 10.1371/journal.pone.0025346

**Published:** 2011-10-17

**Authors:** Tae Woo Kim, Younghye Moon, Kyungjin Kim, Jeong Eun Lee, Hyun Chul Koh, Im Joo Rhyu, Hyun Kim, Woong Sun

**Affiliations:** 1 BK21 Program, Department of Anatomy, Korea University College of Medicine, Sungbuk-Gu, Seoul, Korea; 2 Department of Biological Sciences, Seoul National University, Seoul, Korea; 3 Department of Pharmacology, College of Medicine, Hanyang University, Seoul, Korea; Thomas Jefferson University, United States of America

## Abstract

Parkinson's disease (PD) is a common, late-onset movement disorder with selective degeneration of dopaminergic (DA) neurons in the substantia nigra (SN). Although the neurotoxin 6-hydroxydopamine (6-OHDA) has been used to induce progressive degeneration of DA neurons in various animal models of PD, the precise molecular pathway and the impact of anti-apoptotic treatment on this neurodegeneration are less understood. Following a striatal injection of 6-OHDA, we observed atrophy and progressive death of DA neurons in wild-type mice. These degenerating DA neurons never exhibited signs of apoptosis (i.e., caspase-3 activation and cytoplasmic release of cytochrome C), but rather show nuclear translocation of apoptosis-inducing factor (AIF), a hallmark of regulated necrosis. However, mice with genetic deletion of the proapoptotic gene Bax (Bax-KO) exhibited a complete absence of 6-OHDA-induced DA neuron death and nuclear translocation of AIF, indicating that 6-OHDA-induced DA neuronal death is mediated by Bax-dependent AIF activation. On the other hand, DA neurons that survived in Bax-KO mice exhibited marked neuronal atrophy, without significant improvement of PD-related behavioral deficits. These findings suggest that anti-apoptotic therapy may not be sufficient for PD treatment, and the prevention of Bax-independent neuronal atrophy may be an important therapeutic target.

## Introduction

Parkinson's disease (PD) is one of the most common neurodegenerative disorders characterized by a progressive loss of dopaminergic (DA) neurons in the substantia nigra (SN), which leads to the typical symptoms, including tremor, rigidity, and bradykinesia [1], [2], [3], [4]. Although recent advances have been made in defining biochemical events underlying PD, a precise mechanism of DA neuronal death in PD pathology is yet to be elucidated. In a neurotoxin-induced animal model of PD, large bodies of studies support the apoptotic nature of DA neuronal death. Neuronal apoptosis is mediated by the activation of the pro-apoptotic Bcl-2 family protein Bax, and mitochondrial translocation of Bax triggers the release of cytochrome C into the cytosol, which promotes apoptosome formation and subsequent caspase-3 activation [5], [6]. Intraperitoneal injection of the neurotoxin 1-methyl-1,2,3,6-tetrahydropyridine (MPTP) immediately induced cytoplasmic release of cytochrome C, cleavage of caspase-3, and subsequent increase in terminal deoxynucleotidyl transferase dUTP nick end labeling (TUNEL) [7], [8], [9], [10]. Supporting this, genetic deletion of the pro-apoptotic gene Bax (Bax-KO), or overexpression of the anti-apoptotic gene Bcl-2, suppressed MPTP-induced DA neuronal death [11], [12]. Although these lines of evidence implicate apoptosis, other lines of evidence also suggest the involvement of other types of cell death, such as autophagy and necrosis [13], [14], [15], [16].

Whereas MPTP treatment or administration of neurotoxin 6-hydroxydopamine (6-OHDA) into the SN or medial forebrain bundle quickly induce DA neuronal death within 1 week [12], [17], striatal injection of the 6-OHDA evokes delayed and progressive DA neuronal degeneration (>6weeks), which resembles the human PD pathology [2], [18], [19]. It is far less certain which mechanism of progressive DA neuronal death is induced by striatal injection of 6-OHDA. Although apoptotic profiles based on morphological assessment and TUNEL labeling have been observed in the rat SN following 6-OHDA lesion [20], [21], [22], [23], results from other groups did not support this finding [24], [25]. This is partly attributable to the ambiguity of markers for the identification of the specific types of cell death. For instance, although TUNEL has been widely used as a hallmark for apoptosis, it in fact also detects necrotically degenerating cells [26]. Furthermore, Bcl-2 family molecules not only affect caspase-dependent apoptosis, but also other types of cell death [27]. Accordingly, knockout of the pro-apoptotic gene Bax or overexpression of Bcl-2 proteins can prevent some types of necrosis [28].

In this study, we identified that progressive DA neuronal death induced by striatal injection of 6-OHDA is not dependent on caspase-3 activation, but mediated by the pro-apoptotic gene Bax and apoptosis-inducing factor (AIF). However, 6-OHDA-induced DA neuronal atrophy and early phases of neurodegeneration were also observed in Bax-KO mice. In addition, there was no significant improvement of PD-related behavioral deficits, suggesting that anti-apoptotic therapy may not be sufficient, and that prevention of Bax-independent neuronal atrophy is an important therapeutic target.

## Results

### Effect of striatal injection of 6-OHDA on DA neuronal degeneration in wild-type (WT) and Bax-KO mice

**Figure 1:**
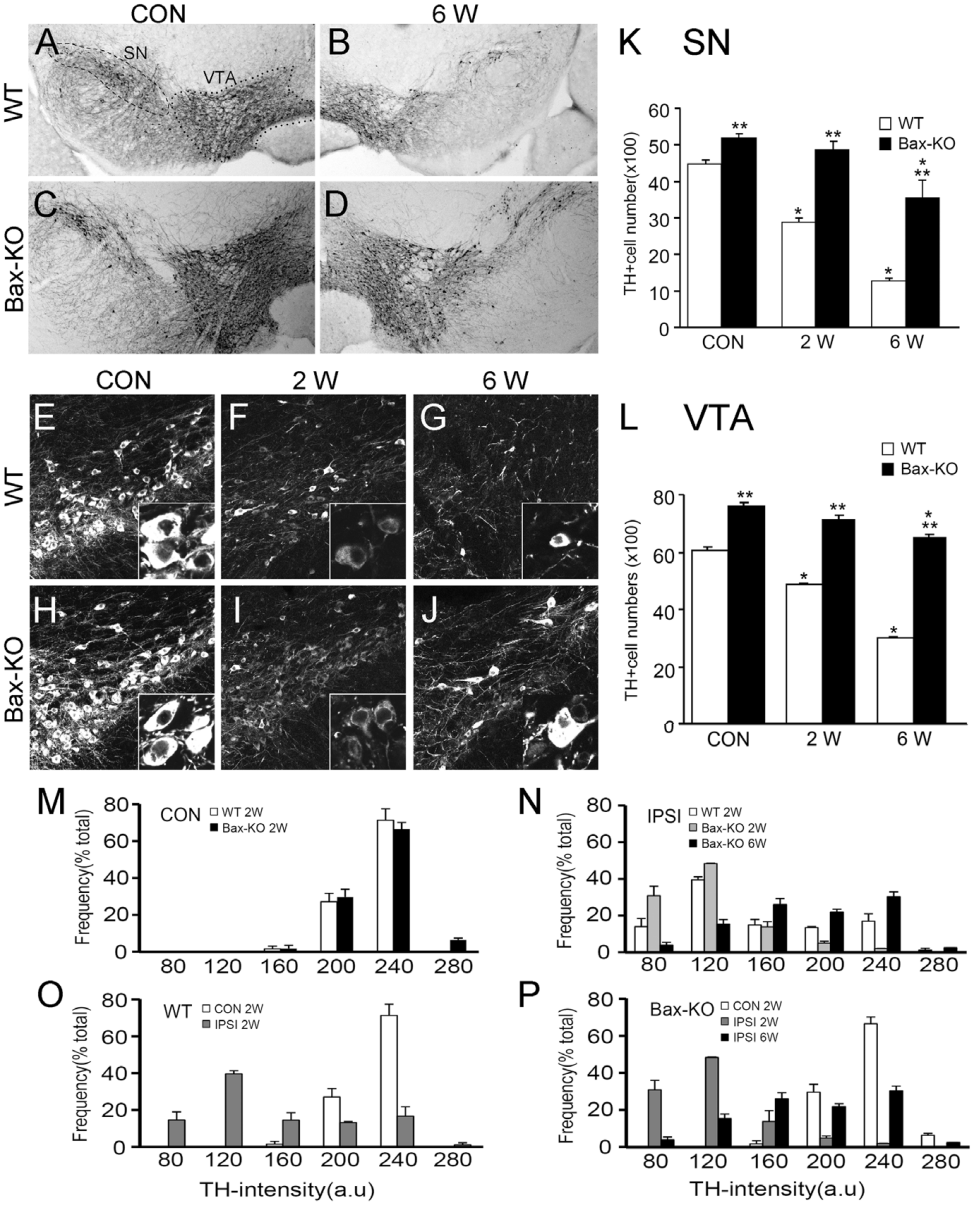
6-OHDA-induced DA neuronal death in wild-type (WT, A–C) and Bax-KO (D–F) mice. **A–D**: WT (**A,B**) or Bax-KO (**C,D**) mice were sacrificed 6 weeks after striatal injection of 6-OHDA, and dopaminergic (DA) neurons in the control (**A,C**) and ipsilateral (**B,D**) midbrain were visualized by immunolabeling of tyrosine hydroxylase (TH). Dashed line indicates in **A** substantia nigra (SN) and dotted line indicates ventral tagmental area (VTA). **E–J**: DA neurons in the SN 2 weeks (**F,I**) or 6 weeks (**G,J**) after striatal injection of 6-OHDA. Scale bar = 50 µm. Controls (CON, **E,H**) show TH immunolabeling in the contralateral side of the SN 6 weeks after 6-OHDA injection. Insets show large magnification images of TH-labeled cells. **K,L**: Quantification of TH+ cells in the SN (**K**) and VTA (**L**) of WT and Bax-KO mice. Data are expressed as mean±s.e.m. n = 4–5, *P<0.05 in Student's t test comparison with control (CON) vs. ipsilateral (IPSI) sides, and **P<0.05 in Student's t-test comparison with WT vs. Bax-KO mice. **M,N**: Comparison of the intensity of THimmunoreactivity between 2-weeks WT (open bars) vs. 2- (grey bars) or 6-weeks (black bars) Bax-KO groups in CON (**M**) and IPSI (**N**) sides. **O,P**: Comparison of THimmunoreactivity between CON and IPSI sides in 2-weeks WT (**O**) vs. 2- and 6- weeks Bax-KO (**P**) groups.

To test the contribution of Bax-dependent DA neuronal death in 6-OHDA-induced PD mouse model, we first explored the extent of DA neuronal death in WT and Bax-KO mice (Fig. 1). Following striatal injection of 6-OHDA in WT mice, there was a progressive decrease in the number of TH+ DA neurons in the midbrain. Sterological quantification of DA neurons in the substantial nigra (SN) and ventral tagmental area (VTA) demonstrated that the numbers of DA neurons in Bax-KO mice were significantly greater than those in WT mice, suggesting that developmental programmed cell death of DA neurons are dependent on the function of Bax gene. The reduction of TH+ neurons was evident in 2 weeks after 6-OHDA injection, and at 6 weeks, approximately 50% (VTA) and 70% (SN) of TH+ neurons disappeared in WT mice (Fig. 1K, L). However, Bax-KO mice did not exhibit significant DA neuronal death in 2 weeks after 6-OHDA treatment, and 90% (VTA) and >60% (SN) of TH+ neurons survived 6 weeks after treatment (Fig. 1K, L), suggesting that DA neuronal death after 6-OHDA treatment is mediated by a Bax-dependent pathway.

Although we found that most Bax-KO DA neurons survived for up to 2 weeks after 6-OHDA treatment, these surviving neurons appeared to have reduced TH expression, similar to WT mice (Fig. 1E vs. 1F and 1H vs. 1I, insets). Quantification of TH-immunoreactivity confirmed this visual impression. DA neurons in WT and Bax-KO contralateral sides exhibited a similar distribution of TH-intensity (Fig. 1M), and a similar reduction in TH-intensity was found in both WT and Bax-KO ipsilateral sides at 2 weeks after 6-OHDA injection (Fig. 1N–P). Our finding implies that some aspects of neurodegeneration (such as neuronal atrophy and loss of TH expression) are dissociated from the Bax-dependent cell death pathway, and thus occurred normally in Bax-KO mice. Supporting this idea, there was a significant reduction in soma size within 2 weeks after 6-OHDA injection both in WT and Bax-KO mice (Fig. S1). On the other hand, a significant fraction of TH+ neurons in 6 weeks-old Bax-KO mice exhibited a partial recovery of TH-intensity and soma size (Fig. 1J, inset, Fig. 1N,P and Fig. S1), suggesting that a subset of Bax-KO-rescued DA neurons restored TH expression.

### Absence of 6-OHDA-induced DA neuronal death in Bax-KO mice

**Figure 2:**
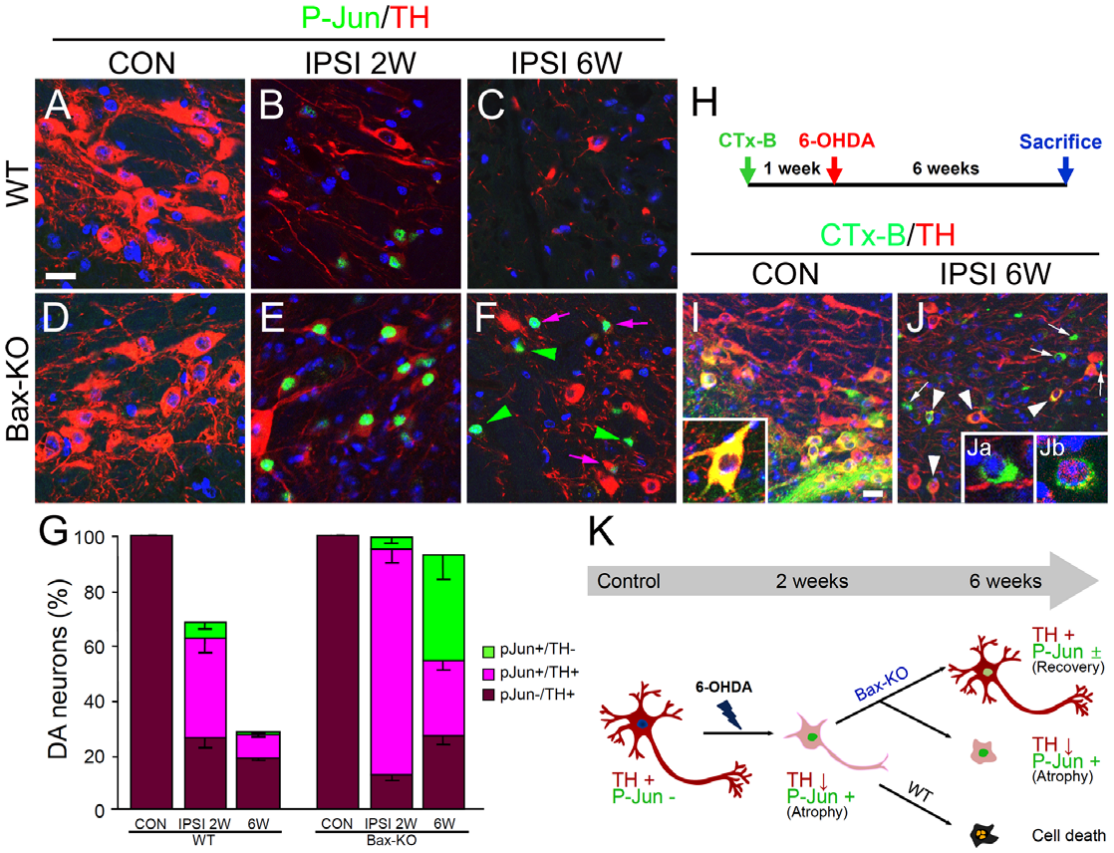
Complete prevention of DA neuronal death in Bax-KO mice. **A–D**: Immunofluorescence labeling of TH (red) and phosphorylated c-Jun (P-Jun, green) in 2-weeks CON (**A,D**), 2-weeks IPSI (**B, E**), and 6-weeks IPSI (**C,F**) sides of WT (**A–C**) and Bax-KO (**D–F**) mice. Nuclei were counterstained with Hoechst33342 (blue). Scale bar = 20 µm. Green arrowheads indicate P-Jun+/TH− cells, and pink arrows indicate P-Jun+/TH+ cells. **G**: Quantification of the proportion of P-Jun- and/or TH-labeled cells in the SN of WT and Bax-KO mice. Data are expressed as mean±s.e.m. n = 3, **H**: Experimental scheme. Alexa 488-conjugated tracer choleratoxin B subunit(Ctx-B) was injected 1-week prior to striatal 6-OHDA injection. Animals were sacrificed 6 weeks after 6-OHDA injection, and retrograde-labeled cells in the SN were examined. **I,J**: Retrograde tracing with CTx-B(green) and immunofluorescence labeling of TH (red) in CON (**I**) and IPSI (**J**) sides of Bax-KO mice. Arrows in **J** indicate theCTx-B-labeled, but TH-negative (CTB+/TH−) cells. Arrowheads indicate the CTx-B-labeled, and TH-expressing cells. Inset in **I** shows normal CTx-B-labeled TH+ cells with dendritic processes. Insets show the higher magnification image of CTx-B+/TH− cells (**Ja**) and CTx-B+/P-Jun+ cells (**Jb**). Note these cells have reduced soma size and reduced/absence of dendritic processes, which are hallmarks of atrophic modification. Nuclei were counterstained with Hoechst33342 (blue). Scale bar = 20 µm. **K**: Summary for the fate of DA neurons in WT and Bax-KO SN after 6-OHDA treatments. Both WT and Bax-KO DA neurons atrophied, with reduced TH expression and induction of P-Jun at early phases of neurodegeneration (2 weeks). Whereas most DA neurons in WT mice underwent cell death within 6 weeks after 6-OHDA injection, virtually all Bax-KO DA neurons survived and exhibited segregation into normally recovered DA neurons (with TH expression and no/reduced P-Jun) and severely atrophied DA neurons (with no TH expression and the maintenance of P-Jun).

Considering that Bax-KO DA neurons progressively lost TH expression after 6-OHDA treatment, it was unclear whether a subset of DA neurons ultimately underwent cell death in a Bax-independent manner, or whether they survived but completely lost TH expression. To address this issue, we tested the phosphorylation of c-Jun (P-Jun) in ipsilateral DA neurons. Previously, we demonstrated that P-Jun was maintained in the surviving, but atrophied, neurons rescued from programmed cell death during development and after nerve injury in Bax-KO mice [29], [30]. Following 6-OHDA injection, many TH+ neurons exhibited enhanced phosphorylation of c-Jun in WT mice by 2 weeks (Fig. 2B), but P-Jun+ cells were barely detected in the 6-weeks-old SN (Fig. 2C), which may be due to the death of WT DA neurons. On the other hand, many P-Jun+ cells were found in the Bax-KO SN 2 and 6 weeks after 6-OHDA treatment (Fig. 2D–F). A subset of these P-Jun+ cells did not exhibit TH immunoreactivity (Fig. 2F, green arrowheads), suggesting that these are surviving DA neurons that completely lost TH expression. When P-Jun+/TH− cells were included in the number of surviving DA neurons, it appeared that most DA neurons survived for up to 6 weeks after 6-OHDA injection in Bax-KO mice (Fig. 2G: P-Jun+/TH−, *F*_(3, 10)_ = 11.945, *P*<0.01). To confirm this, we injected an Alexa 488-conjugated retrograde tracer (Cholera toxin subunit B, CTx-B) into the striatum 7 days prior to 6-OHDA injection, and the SN was monitored 6 weeks after 6-OHDA treatment (Fig. 2H–K). On the contralateral side, most (>97%) CTx-B-labeled cells were TH+ DA neurons in both WT (data not shown) and Bax-KO mice (Fig. 2I). However, a substantial number of retrograde-labeled cells failed to exhibit TH expression in Bax-KO mice (Fig. 2J), suggesting that these DA neurons had innervated the striatum before 6-OHDA injection. Furthermore, most of CTx-B-labeled cells in Bax-KO mice exhibited strong P-Jun immunoreactivity (Fig. 2Jb). These results suggest that DA neurons progressively undergo atrophic (reduced soma size, reduced TH expression and increased P-Jun) alterations before the actual death caused by 6-OHDA injection (2 weeks), which is similar to that seen in Bax-KO mice. These atrophied neurons in WT mice ultimately undergo cell death during a 2- to 6-weeks period, but Bax-KO appears to completely rescue these DA neurons from cell death. However, a subset of DA neurons (∼50%) recovered their TH expression, whereas a subset of neurons underwent extensive atrophy, with marked shrinkage of soma size and complete loss of TH expression (Fig. 2K).

### DA neuronal death induced by 6-OHDA is a caspase-independent, but AIF-mediated, process

**Figure 3:**
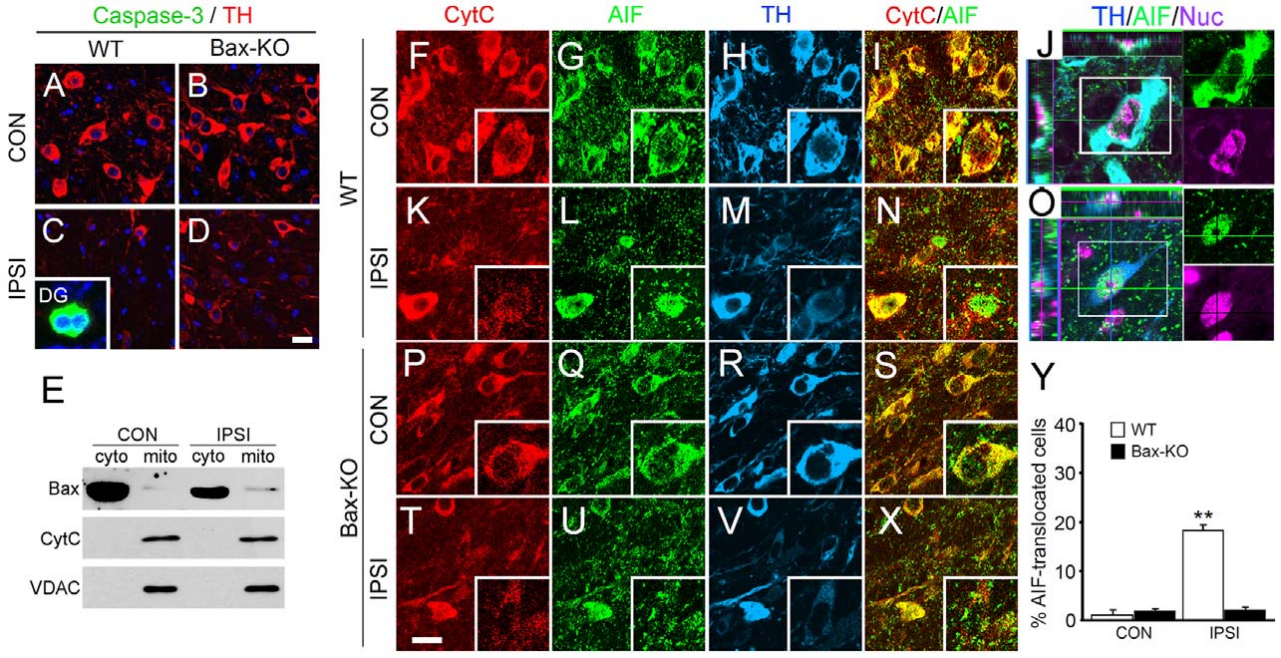
AIF-mediated DA neuronal death following striatal 6-OHDA injection. **A–D**: Immunofluorescence labeling of activated caspase-3 (green) and TH (red) in CON (**A, B**) and IPSI (**C, D**) SN of WT (**A, C**) and Bax-KO (**B,D**) mice 2 weeks after 6-OHDA injection. Inset in **C** shows activated caspase-3 immunoreactive cells in the dentate gyrus (DG) of WT mice. Nuclei were counterstained with Hoechst33342 (blue). Scale bar = 20 µm. **E**: Western blot analysis of subcellular distribution of Bax and cytochrome C 2 weeks after 6-OHDA injection. Cytosolic and mitochondrial fractions were isolated from micropunched SN area from control and ipsilatral sides.**F–O**: Triple immunofluorescence labeling of cytochrome C (**F, K, P, T**, red), AIF (**G, L, Q, U**, green), and TH (**H, M, R, V**, blue) in CON (**F–I, P–S**) and IPSI (**K–N, T–X**) sides of WT (**F–N**) and Bax-KO (**P–S**) SN. **J** and **O** show the z-stacked images demonstrating cytosolic (**J**) vs. nuclear localization (**O**) of AIF. Nuclei were counter-stained with Hoechest33342 and pseudo-colored in purple. Merged images of cytochrome C and AIF in WT (**I,N**) and Bax-KO (**S,X**) SN are shown. Insets demonstrate the higher magnification images. **Y**: Quantification of the DA neurons exhibiting nuclear-translocated AIF signals in the SN. Data are expressed as mean±s.e.m. n = 4, *P<0.01 in Student's t-test comparison.

Whereas Bax-dependent cell death is generally considered as caspase-dependent apoptosis, we were not able to detect any activated caspase-3-labeled cells in a minimum of 20 sections containing the SN from WT and Bax-KO (n = 3–4/group) mice (Fig. 3A–D). Because we occasionally identified activated caspase-3-labeled, spontaneously dying adult-produced neurons in the WT dentate gyrus (DG, inset of Fig. 3C), the absence of activated caspase-3 labeling in the ipsilateral SN is not attributable to an artifact of staining. By mitochondrial fractionation and subsequent western blot analysis, we also failed to monitor cytosolic release of cytochrome C, while Bax was partially translocated to the mitochondria in WT (Fig. 3E). Therefore, we asked whether other forms of neuronal death, such as necrosis and autophagy, are involved in 6-OHDA-induced, Bax-dependent DA neuronal death. Although autophagy has been implicated in the DA neuronal death in some models [31], we failed to detect signs for autophagy, such as LC3 conversion and Beclin1 induction (data not shown), we found that about 20% of TH+ cells exhibited translocation of AIF from the mitochondria to the nucleus in the ipsilateral WT SN, whereas AIF-immunoreactivity was predominant in the cytoplasm/mitochondria of the control side (Fig. 3F–X, Y). All AIF-translocated DA neurons exhibited reduced levels of TH expression, indicating that they were degenerating neurons. Interestingly, these WT DA neurons with nuclear AIF signals maintained cytochrome C immunoreactivity in the mitochondria (Fig. 3K, L). Because release of cytochrome C from mitochondria is an essential step for apoptosis, it appears that 6-OHDA treatment selectively triggered the release of AIF. In Bax-KO mice, however, nuclear translocation of AIF was absent, suggesting that Bax is necessary for AIF activation.

### Partial recovery of striatal DA innervation in Bax-KO mice

**Figure 4:**
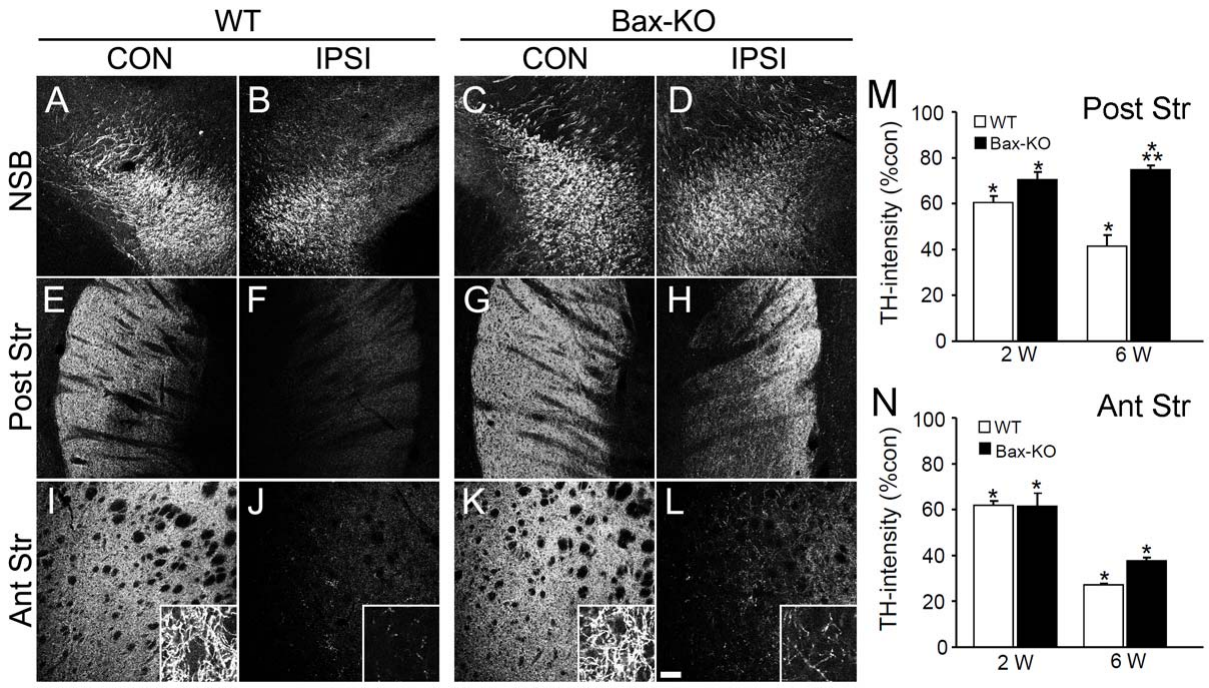
Striatal innervation of TH-immunoreactive DA fibers 6 weeks after 6-OHDA treatment. Distribution of DA fibers were examined at the nigrostriatal bundle (NSB, **A–D**), posterior striatum (Post Str, **E–H**), and anterior striatum (Ant Str, **I–L**) of CON (**A, C, E, G, I, K**) and IPSI (**B, D, F, H, J, L**) sides of WT (**A, B, E, F, I, J**) and Bax-KO (**C, D, G, H, K, L**) mice. Insets in the **I–L** demonstrate the higher magnification images. Scale bar = 100 µm. **M, N**: Quantification of TH intensities on 2 weeks and 6 weeks after 6-OHDA treatment at the posterior striatum (**M**) and anterior striatum (**N**) of WT and Bax-KO mice. Data are expressed as mean±s.e.m. n = 3, *P<0.05 in Student's t test comparison with control (CON) vs. ipsilateral (IPSI) sides, and **P<0.05 in Student's t-test comparison with WT vs. Bax-KO mice.

Because at least a subset of DA neuronal cell bodies recovered TH immunoreactivity at 6 weeks of 6-OHDA treatment in Bax-KO mice, we next tested whether these DA neurons exhibited striatal innervation (Fig. 4). In the ipsilateral side of WT brains, the intensity of TH-labeling was progressively reduced in all anatomical levels we examined, including the nigrostriatal bundle (NSB), posterior striatum (proximal to the DA cell bodies), and anterior striatum (distal to the DA cell bodies; Fig. 4B,F, and J). The TH levels were similarly reduced at 2 weeks after 6-OHDA treatments, whereas Bax-KO mice exhibited substantially more TH+ fibers in the ipsilateral side of the NSB (Fig. 4D) and posterior striatum (Fig. 4H) on 6 weeks. Although there was a marked reduction of TH+ fibers in the ipsilateral anterior striatum compared to the contralateral side, large magnification images demonstrated that significantly more amounts of TH+ fibers were maintained in the ipsilateral side of Bax-KO mice, compared to the that of WT mice (Fig. 4L). Quantification of TH-immunoreactivity in posterior (Fig. 4M) and anterior (Fig. 4N) striatum demonstrated the spared TH+ fiber innervations in the posterior striatum in Bax-KO mice on 6 weeks, suggesting that surviving DA neurons in Bax-KO mice restored their striatal innervation, especially in the posterior striatum.

We also measured the DA contents in the striatum 6 weeks after 6-OHDA. Total DA levels (ng/mg protein) in the control sides were similar in WT and Bax-KO mice (WT, 612±88; Bax-KO, 520±92, t(5) = 0.7003, P = 0.515 in Student's t-test). While DA levels in the ipsilateral side of WT was under the detection limit in our measurements, small but significant level of DA was monitored in the ipsilateral side of Bax-KO mice (35±10, n = 4), suggesting that DA innervation is partially spared in Bax-KO mice.

### Bax-KO does not prevent striatal degeneration and functional deficits

**Figure 5:**
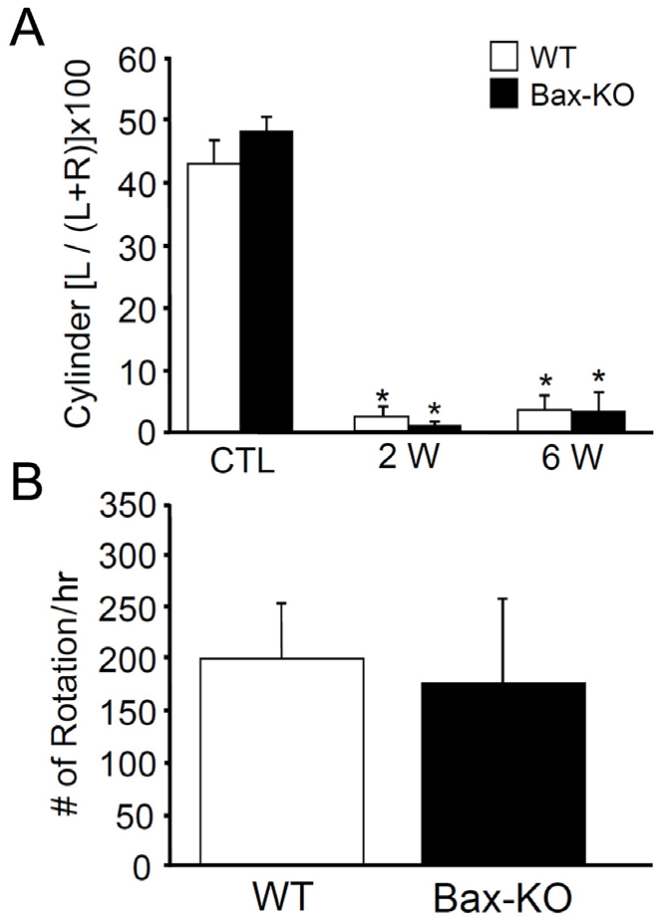
Absence of PD-related behavioral improvement in Bax-KO mice. **A**: Cylinder test. At 2 and 6 weeks after 6-OHDA injection, the rate of ipsilateral forelimb use was measured in WT (n = 6) and Bax-KO (n = 4) mice. **B**: Apomorphine rotation test. The total scores of ipsilateral rotation in 1 hr were scored in WT and Bax-KO mice. Tests were done 6 weeks after 6-OHDA treatment. Data are expressed as mean±s.e.m, n = 6 (WT), n = 4 (Bax-KO).

Although substantially more amounts of striatal innervation of DA neurons were found in Bax-KO mice, Bax-KO mice did not exhibit functional improvements in two different behavioral tests assessing PD-related neurological impairments (Fig. 5). Because 6-OHDA-induced nigrostriatal degeneration promotes asymmetric use of limbs, the cylinder test has been used to assess unilateral forelimb akinesia [32], [33]. In this test, both WT and Bax-KO mice showed a similar time course of forelimb use asymmetry (Fig. 5A). The apomorphine rotation test [32] also showed a similar extent of functional impairment of the nigrostriatal system in WT and Bax-KO mice (Fig. 5B). Collectively, these results suggest that increased survival of DA neurons by Bax deletion had no functional benefits.

## Discussion

In this study, we provide evidence that Bax is indispensable for the 6-OHDA-induced DA neuronal death, and virtually all Bax-KO DA neurons survived until 6 weeks after 6-OHDA treatment. Although all DA neurons were rescued from cell death in Bax-KO mice, a large subset (>40%) of surviving neurons at 6 weeks maintained their atrophied morphology, such as reduction in soma size, loss of TH expression, and increased P-Jun. Previously, we have characterized that the absence of trophic signals or neuronal damage triggered P-Jun, which is an upstream event for Bax activation [29]. Furthermore, P-Jun was also increased in dying rat DA neurons following striatal 6-OHDA injection [34]. Therefore, we employed P-Jun as a marker for atrophied neurons. Interestingly, neuronal atrophy was similar in both WT and Bax-KO DA neurons at early phases (2 weeks after 6-OHDA injection) of neurodegeneration, suggesting that DA neuronal atrophy occurs independently of the Bax-dependent cell death pathway, and the blockade of neuronal death by Bax deletion is not sufficient for preventing neuronal atrophy. In fact, dissociation of neuronal death and atrophy is commonly observed in Bax-KO mice during development [35], [36], after nerve axotomy [30], [37], and in an amyotrophic lateral sclerosis model [38], indicating that the neuronal death and neuronal atrophy are mediated by two independent mechanisms.

By 6 weeks after 6-OHDA injection, most WT DA neurons had degenerated, and accordingly, few P-Jun-labeled neurons remained. However, DA neurons in Bax-KO mice appeared to be segregated into two distinct populations. A subset (∼50%) of DA neurons completely lost TH expression and exhibited a strong P-Jun expression. Others exhibited substantial recovery of TH expression, but lost P-Jun. Similar segregation of surviving neurons was previously found in developing motoneurons (MNs) of Bax-KO mice [36], [39]. During development, usually 50% of initially produced MNs undergo Bax-dependent programmed cell death, and thus Bax-KO mice maintain a two-fold increase in the number of surviving MNs than that of their WT littermates [36]. However, these surplus MNs did not maintain innervation to the target muscle, and exhibited severe atrophic modifications, including reduced cell size and P-Jun expression, and loss of choline acetyltransferase (ChaT) expression. By elimination of surplus MNs from the target connection, normal numbers of MNs innervated the target muscle, and Bax-KO mice did not exhibit motor-related deficits [29], [36]. We do not believe that this segregation is owing to the molecular differences because increase in the neurotrophic signals restored innervating ability of atrophied MNs [37]. Similarly, we found that only a subset of DA neurons restored the striatal innervation, and differential ability of target (striatal) innervation of surviving DA neurons may be involved in the segregation process, although the precise mechanism is required to be elucidated in the future.

Although most forms of Bax-dependent cell death involve caspase-dependent apoptosis, here we found that striatal injection of 6-OHDA promoted Bax-dependent, but caspase-independent DA neuronal death. Neither caspase-3 activation nor cytochrome C release was found in degenerating DA neurons (i.e., atrophied neurons with marginal TH expression) after 6-OHDA injection. Because we infrequently observed activated caspase-3 labeling in spontaneously degenerating, adult-generated neurons in the DG in the same section, we believe that the absence of activated caspase-3 labeling in DA neurons was not due to poor staining or simply missing the labeled cells. Although previous reports demonstrated caspase-3 activation in a mouse model of PD with MPTP treatment [40], [41], [42], human PD brain samples [43], [44], and *in vitro* models with 6-OHDA [16], [45], [46], [47], there is no report demonstrating caspase-3 activation in a mouse or rat model of striatal 6-OHDA injection *in vivo* [25]. Therefore, progressive DA neuronal death induced by striatal injection of 6-OHDA does not appear to require caspase-3 activation.

Our current observations suggest that DA neuronal death is mediated by the AIF pathway, because degenerating DA neurons in WT mice exhibited nuclear translocation of AIF. AIF is a mitochondrial flavoprotein that mediates caspase-independent cell death [48], [49], [50], [51]. In healthy cells, mitochondrial AIF may protect against oxidative stress, but once released into the cytosol, AIF is translocated to the nucleus where it directly induces chromatin condensation and chromatolysis in a caspase-independent manner. The release of AIF is modulated by mitochondrial outer membrane permeabilization (MOMP), which is partly regulated by the Bcl-2 family proteins, including Bax [52], [53]. Bax can alter mitochondrial membrane permeability by forming pores after oligomerization, or modulating existing channels like VDAC [54], [55], and modulation of Bcl-2/Bax dictates cellular fate by increasing mitochondrial outer membrane permeability and AIF release [52], [56]. In this study, we found that AIF translocation was completely absent in Bax-KO mice, indicating that AIF translocation following 6-OHDA-induced DA neuronal death is Bax-dependent. Increasing evidence supports a prominent role of AIF in neurodegeneration, including PD. AIF translocation occurs after toxic insults, including traumatic brain injury [57], oxidative stress [58], cerebral ischemia [59], Alzheimer's disease [60], and MPTP-induced PD models [13]. Recently, nuclear translocation of AIF was also found in the degenerating DA neurons in autopsies of human PD patients, suggesting that AIF activation plays a significant role in the DA neuronal death in PD patients [56].

It appears that AIF is preferentially released from the mitochondria via a Bax-induced mitochondrial pore, because AIF-translocated WT neurons maintained mitochondrial cytochrome C immunoreactivity. Similarly, AIF release was detected before the cytochrome C release during NMDA (N-methyl-D-aspartate)-induced neuronal death [61], [62], [63]. It is known that AIF is localized in the intermitochondrial space, and can be released by calpain-dependent protein cleavage [64], [65]. Some AIF proteins are also localized in the outer mitochondrial membrane on the cytosolic side [66], and may be released without calpain activation [67]. On the other hand, cytochrome C is anchored to the mitochondrial membrane by conjugation with the mitochondrial membrane lipid cardiolipin [68], [69]. Therefore, cytochrome C release is dependent on the peroxidation of cardiolipin [70], and distinct anchorage and release mechanisms of AIF and cytochrome C may explain the preferential release of AIF via a Bax-induced mitochondrial pore. Furthermore, it is known that expression of Apaf-1 is down-regulated in mature neurons, making neurons difficult to form apoptosome under the low concentration of cytosolic cytochrome C [71]. Therefore, similar mechanisms may also contribute to the selective activation of AIF pathway in progressively degenerating DA neurons.

Although Bax-KO mice exhibited a complete absence of DA neuronal death and partial restoration of DA fibers in the striatum 6 weeks after 6-OHDA treatment, there was no significant improvement in PD-related neurological deficits in Bax-KO mice. It is known that intrastriatal supplement of neurotrophic factors, such as glial cell-derived neurotrophic factor (GDNF), is more potent than the nigral supplement for the regeneration of striatal DA fibers and functional recovery, although nigral GDNF delivery was more potent for the survival of DA neurons from 6-OHDA-induced neurodegeneration [72], [73]. Because Bax deletion primarily affects the survival of DA neuronal cell body, insufficient regeneration/recovery at the striatal level may cause the failure in the functional improvements. Supporting this, we found that DA contents in Bax-KO ipsilateral striatum was markedly reduced, although it was higher than WT littermates. Furthermore, we currently do not know whether DA fibers in the Bax-KO ipsilateral striatum are functional and efficiently releasing DA, and thus we cannot completely rule out the possibility that not only the number, but also the quality of DA fibers in Bax-KO mice are insufficient for the functional recovery.

In conclusion, our present study provides evidence that striatal injection of 6-OHDA induces Bax-dependent, AIF-mediated DA neuronal death. However, neuronal atrophy appears to be a Bax-independent mechanism, and Bax-KO mice did not exhibit behavioral benefits. These results demonstrate the potentials and limitations of a therapeutic strategy for anti-Bax treatment of PD.

## Materials and Methods

### Animals and surgery

Bax-KO mice were obtained from Jackson Laboratory (Bar Harbor, ME), and maintained as heterozygotes on a C57BL/6 background. Homozygous mice were obtained by breeding heterozygote male and female mice, and the genotypes of the sibling animals were individually assessed by polymerase chain reaction (PCR) as previously described [74]. For stereotaxic injection of neurotoxin, adult male mice (25–30 g) were deeply anesthetized with sodium pentobarbital (50 mg/kg) and placed in a stereotaxic device (Stoelting, Wood Dale, IL). Neurotoxin, 6-OHDA (15 µg in 1.5 µl of PBS containing 0.02% ascorbate), was injected into the right striatum (anterior, +0.05 mm; medial, −0.2 mm; dorsal, −0.35 mm relative to bregma) by a 30-gauge microsyringe at a rate of 0.5 µl/min. After a pause for 15 min, the needle was slowly withdrawn.

For retrograde tracing, some animals received bilateral injection of 1 µl retrograde fluorescent tracer Alexa Fluor 488-conjugated Cholera Toxin Subunit B (CTx-B; Molecular probes, Eugene, OR) into the striatum (anterior, +0.06 mm; medial, −0.22 mm; dorsal, −0.35 mm relative to bregma) 7 days before a 6-OHDA injection. All experiments were carried out in strict accordance with the ethical guidelines of Korea University, and with the approval of the Animal Care and Use Committee of Korea University (Permit Number: KUIACUC-2011-127).

### Histology

For immunohistochemical analysis, mice were perfused with 4% paraformaldehyde and the brains were isolated. Following post-fixation in the same fixative overnight, brains were cryoprotected in 30% sucrose, sectioned serially (40 µm), and stored in 50% glycerol/50% PBS solution at−20°C until use. Every 6^th^ or 12^th^ section containing the SN was blocked with 3% BSA in PBS for 30 min. Primary antibodies were then applied to the sections overnight at room temperature. Primary antibodies used in this study were a rabbit polyclonal tyrosine hydroxylase (TH; 1∶1000; Millipore, Temecula, CA), a mouse monoclonal TH (1∶500; Sigma, St. Louis, MO), rabbit polyclonal phosphorylated-c-Jun (1∶250; Cell Signaling, Beverly, MA), sheep cytochrome c (1∶500; Sigma), rabbit monoclonal AIF (1∶250; Epitomics, Burlingame, CA), and activated caspase-3 (1∶250; Millipore). After several washes with PBS, sections were incubated with appropriate secondary antibodies for 30 min. Nuclei were then counterstained with Hoechst33342, and the images were captured with a Zeiss LSM510 confocal microscope. For the measurement of the intensity of TH-immunoreactivity, all images were captured under the identical condition, and the intensity within the cell body or in the striatum was obtained using the ImageJ program (http://rsbweb.nih.gov/ij/).

### Stereological analysis

To quantify the number of TH-positive (TH+) neurons, an optical dissector method of stereological analysis was employed [74]. Using StereoInvestigator Software (MicroBrightField, Willston, VT), a fractionator probe was established for each section, and every 6^th^ section covering the entire SN was examined. The number of TH-positive neurons in each counting frame was then determined by focusing down through the section, using a 40× objective. Our criteria for counting an individual TH+ neuron was the presence of its nucleus within the counting frame, or touching the right or top lines (green), but not touching the left or bottom lines (red). The total number of TH+ neurons in each section of the SN was then mathematically calculated by the Stereo Investigator program.

### Western blotting

For acquirement of SN, brain was rapidly frozen on dry ice, and placed on a cryostat. Coronal slices (0.5 mm) containing SN were taken, and SN region was punched out with 1.0 mm micro-punch. Punched tissues were homogenized in ice-cold homogenization buffer (250 mM sucrose, 10 mM HEPES, 1 mg/ml BSA, 0,5 mM EDTA and 0.5 mM EGTA, pH 7.4) using a hand-held Tefalon glass homogenizer (0.1 ml) by three gentle strokes and centrifuged at 2000×g for 3 min. Supernatant was centrifuged at 12,000×g for 8 min, and the supernatant was taken as a cytosolic faction. The pellet was then washed with sucrose buffer (320 mM sucrose, 3 mM CaCl_2_, 2 mM Mg·acetate, 0.1 mM EDTA, 10 mM Tris-HCl, pH 8.0) and re-centrifuged at 12,000×g for 10 min. The final pellet was resuspended in sucrose buffer (mitochondrial fraction). Same amount (10 µg) of mitochondrial fraction and cytosolic fraction was size-seperated through denaturing SDS-PAGE. Primary antibodies used were a rabbit polyclonal TH (1∶1000; Abcam, Cambridge, MA), a mouse monoclonal cytochrome C (1∶1000; Pharmingen, San Diego, CA), and a goat VDAC (1∶1000; SantaCruz Biotechnonogy, Santa Cruz, CA). The blot was washed, hybridized with appropriate secondary antibodies, and the signal was detected using a Supersignal ChemiLuminiscence detection kit (Pierce, Rockford, IL) as described by manufacturer.

### HPLC

The levels of DA in the brain tissues were determined by a modified method [75]. The tissue was homogenized with a tip in 280 µl of 0.2 M perchloric acid containing 0.1 mM EDTA and 20 µl of 10 µM 3,4-Dihydroxybenzylamine hydrobromide as an internal standard. Cell membranes were disrupted using a handy sonicator (Tomy Seiko, Tokyo, Japan) in an ice bath and spun down at 13,000 rpm for 10 min at 4°C using a centrifuge. The supernatants were filtered through centrifugal filter devices (Microcon YM-10, Millipore Co., Bedford, MA, USA) by centrifuging at 13,000 rpm for 15 min at 4°C. After filtration, 20 µl of the sample was injected directly into an injector (7725i, Rheodyne, Cotati, CA, USA) and analyzed by a high-performance liquid chromatograph (HPLC) with electrochemical detection. The pellets were used for measurements of protein concentration. The HPLC system consisted of an electrochemical detector (ECD-300, EICOM, Kyoto, Japan), a solvent delivery system (515 HPLC-pump; Waters Co., Milford, MA, USA), a column oven (Waters Co., Milford, MA, USA), and a data processor dsCHROM-net (Donam Int., Seoul, Korea). The separation column was a reverse-phase C18 column (3.0 mm i.d.×150 mm; EICOMPAK SC-50DS; EICOM) and the guard column was a PREPAK (4.0 mm i.d.×5 mm, EICOM). The appendance potential of ECD-300 (carbon electrode vs. Ag/AgCl reference electrode) was set at +750 mV. The mobile phase consisted of 90 mM sodium acetate–100 mM citric acid buffer (pH 3.5)/methanol (83∶17, v/v) containing 190 mg/L of sodium-1-octanesulfonic acid and 5 mg/L of 2Na EDTA. The flow rate was set at 0.5 ml/min at 30°C. 3,4-Dihydroxybenzylamine hydrobromide was used as the internal standard for the quantification of DA concentrations. DA levels were calculated using DA standard (3-hydroxydopamine; dopaminehydrochloride) and corrected by protein levels of the samples. The levels of DA were expressed as ng/mg protein.

### Behavioral tests

Preference of forelimb use during explorative activity was analyzed in the cylinder test [33]. The test was performed from 1 to 6 weeks after 6-OHDA injection. Mice were placed individually in a glass cylinder (11-cm diameter, 30-cm height) for 10 min, and an experimenter who was blinded to the group counted the number of wall contacts and landing contacts made with the forepaws. The preference of the forelimb use was then scored by the following equation: [L/(L+R)]×100, where L is the number of left forelimb use, and R is the number of right forelimb use during the test periods.

For rotation tests, mice were given 0.5 mg/kg apomorphine6weeks after 6-OHDA injection. Immediately after injection of apomorphine, the animals were individually placed in a test bowl, and the number of rotations made clockwise was monitored for 60 min [32].

### Statistical analysis

Statistical significance of differences between the groups was evaluated by two-tailed (α = 0.05) one-way ANOVA with Scheffe's multiple comparison or independent sample t-tests. All the analyses were carried out with SPSS software, and all values are given as mean ± SEM.

## Supporting Information

Figure S1 Comparison of the soma size of TH immunoreactive DA neurons between 2-weeks WT (open bars) vs. 2- (grey bars) or 6-weeks (black bars) Bax-KO groups in CON (left upper graph) and IPSI (right upper) sides. Comparison of soma sizes between CON and IPSI sides in 2-weeks WT (left lower graph) vs. 2- and 6- weeks Bax-KO (right lower graph) groups. Most Bax-KO DA neurons which were survived 2 weeks after 6-OHDA treatment exhibited the reduction of the soma size, similar to the WT mice. On the other hand, TH+ neurons in 6 weeks-old Bax-KO mice exhibited partial recovery of soma size. Furthermore, small fraction of Bax-KO DA neurons exhibited hypertrophy. For example, the proportion of DA neurons with soma size >480 µm^2^ was significantly increased in 6-weeks Bax-KO IPSI group. This neuronal hypertrophy is known to be associated with regenerative processes after neuronal injury [76], indicating the partial regeneration in Bax-KO mice.
(TIF)
