# Supplementary figures and images for: Dissociation of Progressive Dopaminergic Neuronal Death and Behavioral Impairments by Bax Deletion in a Mouse Model of Parkinson's Diseases
Source: PLoS One. 2011 Oct 17;6(10):e25346. doi: 10.1371/journal.pone.0025346 (PMC3197195; doi:10.1371/journal.pone.0025346)

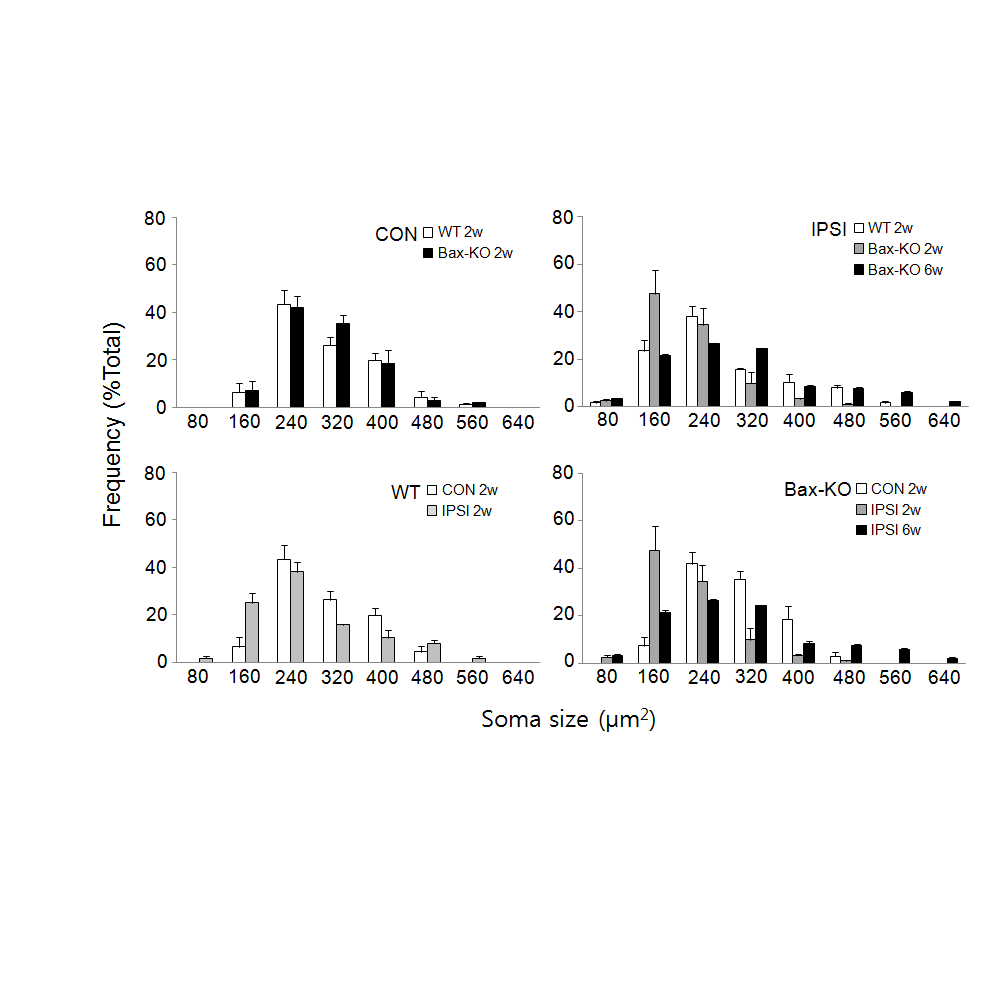

Supplement: Figure S1 — Comparison of the soma size of TH immunoreactive DA neurons between 2-weeks WT (open bars) vs. 2- (grey bars) or 6-weeks (black bars) Bax-KO groups in CON (left upper graph) and IPSI (right upper) sides. Comparison of soma sizes between CON and IPSI sides in 2-weeks WT (left lower graph) vs. 2- and 6- weeks Bax-KO (right lower graph) groups. Most Bax-KO DA neurons which were survived 2 weeks after 6-OHDA treatment exhibited the reduction of the soma size, similar to the WT mice. On the other hand, TH+ neurons in 6 weeks-old Bax-KO mice exhibited partial recovery of soma size. Furthermore, small fraction of Bax-KO DA neurons exhibited hypertrophy. For example, the proportion of DA neurons with soma size >480 µm2 was significantly increased in 6-weeks Bax-KO IPSI group. This neuronal hypertrophy is known to be associated with regenerative processes after neuronal injury [76], indicating the partial regeneration in Bax-KO mice. (TIF) [file pone.0025346.s001.tif]
